# Proteolytic processing of both RXLR and EER motifs in oomycete effectors

**DOI:** 10.1111/nph.20130

**Published:** 2024-09-27

**Authors:** Lin Xu, Shumei Wang, Wei Wang, Haixia Wang, Lydia Welsh, Petra C. Boevink, Stephen C. Whisson, Paul R. J. Birch

**Affiliations:** ^1^ Division of Plant Sciences, School of Life Sciences University of Dundee, at James Hutton Institute Invergowrie Dundee DD2 5DA UK; ^2^ State Key Laboratory of Crop Stress Biology for Arid Areas and College of Plant Protection Northwest Agriculture & Forestry University Yangling Shanxi 712100 China; ^3^ Cell and Molecular Sciences, James Hutton Institute Invergowrie Dundee DD2 5DA UK

**Keywords:** cytoplasmic effector, effector secretion, late blight, pathogenicity, *Phytophthora*, virulence

## Abstract

- Arg‐any amino acid‐Leu‐Arg (RXLR) effectors are central oomycete virulence factors that suppress plant immunity. Relatively little is known about how they are processed post‐translationally before delivery into host cells.
- A range of molecular, cell and biochemical processes were used to investigate proteolytic processing of RXLR and Glu‐Glu‐Arg (EER) motifs in *Phytophthora infestans* effectors.
- Proteolytic cleavage at the RXLR motif occurred before secretion in all effectors tested, suggesting it is a general rule. Cleavage occurred between the leucine and the second arginine. There was no cleavage of a naturally occurring second RXLR motif in a structured region of Pi21388/AvrBlb1, or one introduced at a similar position in effector Pi04314, in keeping with the motif being positionally constrained, potentially to disordered regions closely following the signal peptide. Remarkably, independent proteolytic cleavage of the EER motif, often found immediately after the RXLR, was also observed, occurring immediately after the arginine. Full‐length effectors expressed in host plant *Nicotiana benthamiana* revealed that, although secreted, they were poorly processed, suggesting that RXLR and EER cleavage does not occur in all eukaryotic cells.
- We conclude that, whether possessing both RXLR and EER, or either motif alone, these effectors are likely generally proteolytically processed before secretion.

## Abstract 2

See also the Commentary on this article by Meisrimler & Eccersall, **245**: 1338–1340.

## Introduction

Diseases caused by plant pathogens and pests result in a considerable threat to food security, including up to 23% losses of the five most significant food crops (Savary *et al*., 2019). Amongst the most economically significant disease agents are fungal and oomycete (filamentous) pathogens. The oomycete genus *Phytophthora* includes some of the most devastating plant pathogens (Kamoun *et al*., 2015; Derevnina *et al*., 2016). For example, *Phytophthora infestans*, causing potato and tomato late blight, precipitated the Irish potato famines of the 19^th^ century. It remains the most damaging potato and tomato disease globally (Fry *et al*., 2015; Kamoun *et al*., 2015).

*Phytophthora* spp. secrete ‘effector’ proteins that act either outside (apoplastic effectors) or are delivered to the inside (cytoplasmic effectors) of living plant cells. Prominent amongst cytoplasmic effectors are a class containing the conserved Arg‐any amino acid‐Leu‐Arg (RXLR) motif (Rehmany *et al*., 2005) located closely downstream of the signal peptide. RXLR effectors target multiple proteins and processes at diverse locations inside host cells to suppress immunity (He *et al*., 2020; Fabro, 2021; Petre *et al*., 2021; McLellan *et al*., 2022; Wang *et al*., 2023).

Many filamentous pathogens, including *P. infestans*, form haustoria, hyphal infection structures that are intimately associated with living plant cells. Haustoria are sites of cross‐kingdom molecular exchange and, as such, represent key battle grounds that determine host susceptibility or resistance (Boevink *et al*., 2020; Bozkurt & Kamoun, 2020; King *et al*., 2023). RXLR effectors have been shown to enter plant cells following their unconventional secretion from haustoria. By contrast, although also secreted from haustoria, apoplastic *P. infestans* effectors follow the canonical ER‐to‐Golgi pathway that is sensitive to the inhibitor brefeldin A (BFA) (Wang *et al*., 2017, 2018). Unconventional secretion of cytoplasmic effectors and conventional secretion of apoplastic effectors has also been observed for the fungal pathogen *Magnaporthe oryzae* (Giraldo *et al*., 2013). More recently, it has been reported that *P. infestans* RXLR effectors can be taken into plant host cells via clathrin‐mediated endocytosis (CME) (Wang *et al*., 2023a). Similarly, *M. oryzae* cytoplasmic effectors have also been observed to enter plant cells via CME (Oliveira‐Garcia *et al*., 2023), hinting at a potential universal strategy employed by haustoria‐forming filamentous pathogens (Wang *et al*., 2023b).

The RXLR motif is required for effector translocation into plant cells (Whisson *et al*., 2007). However, its precise role has been difficult to elucidate and has often led to controversy (Ellis & Dodds, 2011; Boevink *et al*., 2020; Bozkurt & Kamoun, 2020). The RXLR motif was reported to bind to phosphoinositide‐3‐phosphate (PI3P) on the outer surface of plant cells, promoting uptake in a pathogen‐autonomous manner (Kale *et al*., 2010). However, pathogen‐independent uptake was drawn into question (Wawra *et al*., 2013; Wang *et al*., 2017), as was the PI3P‐binding of the RXLR motif (Yaeno *et al*., 2011; Wawra *et al*., 2012). Indeed, the RXLR motif has been reported to be a site of proteolytic cleavage before effector secretion (Wawra *et al*., 2017), implying that it is not responsible for binding to PI3P on the outer surface of the plant cell membrane.

Cleavage at the RXLR motif is reminiscent of proteolytic cleavage at an equivalent motif, RXLXE/D/Q, also called the *Plasmodium* export element (PEXEL), in effectors of the malaria parasite, which are delivered into host blood cells (Boddey *et al*., 2010; Russo *et al*., 2010). As is apparent with the RXLR motif, the PEXEL motif is positionally constrained to within 40 amino acids after the signal peptide (SP) cleavage site (Battacharjee *et al*., 2006; Win *et al*., 2007; Win & Kamoun, 2008). Its spatial conservation and rapid proteolytic cleavage, followed by acetylation of the N‐terminus, are important for effector secretion via a specialised export pathway (Boddey *et al*., 2016).

In addition to the RXLR motif, many *Phytophthora* cytoplasmic effectors also contain a conserved Glu‐Glu‐Arg (EER) motif, immediately downstream of the RXLR, which has again been implicated in effector delivery into host cells (Whisson *et al*., 2007). Moreover, a ‘WY’ domain represents a conserved structural fold within the C‐terminal half of many RXLR effectors that contributes to host target specificity (Boutemy *et al*., 2011; Win *et al*., 2012; Bentham *et al*., 2023; Li *et al*., 2023). Interestingly, a number of expressed effectors from *Bremia lactucae* that are predicted to contain the WY structural fold and are recognised by host resistance proteins only contain the EER motif (Wood *et al*., 2020). Given that there are functionally characterised effectors containing only the RXLR motif, this raises the possibility that both motifs are effector processing sites (Wang *et al*., 2023).

In this work, we set out to investigate whether cleavage at the RXLR motif is evident in diverse *P. infestans* effectors. We discovered that cleavage is indeed evident in effectors containing RXLR‐only or RXLR‐EER motifs. Moreover, we observed that cleavage occurs after the leucine in the RXLR motif, rather than the second arginine as previously reported (Wawra *et al*., 2017). We confirm that cleavage of the RXLR is constrained by its location after the SP. Unexpectedly, we discovered that the EER motif is also cleaved after the arginine, revealing that both RXLR and EER are sites for proteolytic processing.

## Materials and Methods

### *Phytophthora infestans* culture and *Nicotiana benthamiana* inoculation

*Phytophthora infestans* (Mont.) de Bary wild‐type (WT) strain 3928A was cultured on rye agar medium (Caten & Jinks, 1968) containing ampicillin (Amp, 50 μg ml^−1^) and pimaricin (Pim, 20 μg ml^−1^). Transgenic lines were cultured on rye agar medium containing Amp, Pim and geneticin (G418, 10 μg ml^−1^) at 19°C in the dark. 10–14‐d‐old *P. infestans* culture plates were use for inoculum preparation and the final concentration was adjusted to 8 × 10^4^ sporangia ml^−1^. Detached and wounded *Nicotiana benthamiana* leaves were inoculated with 10 μl inoculum. Inoculated leaves were incubated on moist tissue in sealed clear plastic boxes at 19°C in the dark for the first 24 h, then under a normal day–night (16 h : 8 h) light regime (Kunjeti *et al*., 2016; He *et al*., 2018).

### Vector construction for *P. infestans* transformation

Coding sequences of Pi09216 (XP_002903512), Pi04314 (XP_002905959), Pi21388/IPIO1/AvrBlb1 (XP_002895051), Pi22926 (XP_002901758), Pi09160 (XP_002903475) and Pi04145 (XP_002905847) were amplified from 3928A genomic DNA using gene‐specific primers containing the NotI restriction enzyme recognition sequence (Supporting Information Table S1). Nested PCR approach was adopted to amplify Pi04097 (XP_002905808) since it is part of the AvrBlb2 gene family (Table S1). PCR products were purified (MinElute PCR Purification Kit; QIAGEN), digested with NotI (New England Biolabs (NEB), Hitchin, UK) and ligated into NotI‐digested and shrimp alkaline phosphatase (NEB) dephosphorylated pPL‐RAG vector (Wang *et al*., 2017) using T4 DNA ligase (NEB).

The RXLR or EER mutant vectors were generated using the QuikChange II XL site‐directed mutagenesis kit (Agilent, Santa Clara, CA, USA). Gene‐specific mutagenic primers were designed using the QuikChange primer design program (https://www.agilent.com/store/primerDesignProgram.jsp) (Table S1). pPL‐RAG‐09216, pPL‐RAG‐04097, pPL‐RAG‐04314, pPL‐RAG‐21388 and pPL‐RAG‐22926 were used as templates to yield the pPL‐RAG‐09216AAAA, pPL‐RAG‐04097AAAA, pPL‐RAG‐04314AAAA‐EER, pPL‐RAG‐04314RXLR‐AAA, pPL‐RAG‐04314RXLR‐EER‐RXLR, pPL‐RAG‐21388AAAA‐EER, pPL‐RAG‐21388RXLR‐AAA, pPL‐RAG‐22926AAAA‐EER and pPL‐RAG‐22926RXLR‐AAA vectors, respectively. Then, pPL‐RAG‐04314AAAA‐EER, pPL‐RAG‐21388AAAA‐EER and pPL‐RAG‐22926AAAA‐EER vectors were used as templates to yield the pPL‐RAG‐04314AAAA‐AAA, pPL‐RAG‐04314AAAA‐EER‐RXLR, pPL‐RAG‐21388AAAA‐AAA and pPL‐RAG‐22926AAAA‐AAA vectors, respectively.

Transformation of *P. infestans* was carried out according to a modified polyethylene glycol (PEG)‐CaCl_2_‐lipofectin mediated method (Judelson *et al*., 1991; Avrova *et al*., 2008). The WT used for transformation was a line regenerated from 3928A protoplasts (3928A mock) that had been through the transformation procedure but without any transforming DNA (mock transformed). The protoplasting solution contained 5 mg ml^−1^ Extralyse (Laffort) and 4 mg ml^−1^ cellulase from *Trichoderma viride* (Duchefa Biochemie, Haarlem, The Netherlands).

### Vector construction for expression *in planta*

Pi09216_1–175_, Pi04314_1–154_, Pi21388_1–152_ and Pi22926_1–198_ (full‐length coding sequences), Pi09216_38–175_, Pi04314_57–154_, Pi21388_55–152_ and Pi22926_47–198_ (coding sequences immediately after the RXLR motif), and Pi04314_68–154_, Pi21388_73–152_ and Pi22926_53–198_ (coding sequences immediately after the EER motif) were amplified using gene‐specific primers containing flanking Gateway (Invitrogen) recombination sequences (Table S1). PCR products were purified and recombined into the pDONR201 entry vector (BP reaction) to generate donor vectors. These donor vectors were recombined with destination vectors pK7RWG2 (LR reaction) to generate overexpression vectors. Sequence‐verified plasmids were electroporated into *Agrobacterium tumefaciens* strain GV3101 for transiently overexpressing mRFP fusion proteins in *N. benthamiana*.

*Agrobacterium tumefaciens* containing overexpression vectors were prepared for plant infiltration as described by Wang *et al*. (2017). The concentration was adjusted to the appropriate optical density at 600 nm (OD600): 0.01–0.05 for confocal imaging and 0.5 for immunoblots.

### Protein extraction and immunoprecipitation

Sporangia of *P. infestans* transformants were collected from 10 to 14‐d‐old plates and inoculated into 5 ml of amended lima bean (ALB) liquid medium (Bruck *et al*., 1980) containing 10 μg ml^−1^ G418 and kept at 19°C in the dark for growth for 3 d, and the mycelium (M) were then transferred to 1 ml of ALB liquid medium containing G418. After 1 d, M were separated from the growth medium (referred to as culture filtrate, CF) and semidried using tissue. The semidried M were thoroughly mixed and heated at 95°C for 10 min with 50 μl 2 × sodium dodecyl sulfate (SDS) loading buffer (Wang *et al*., 2017), centrifuged at 13 000 ***g*** for 10 min, and the supernatant was taken for SDS‐PAGE. The CF was centrifuged at 1000 ***g*** for 5 min to remove cellular debris. Total proteins were precipitated using chloroform/methanol precipitation (Fic *et al*., 2010). Briefly, four volumes of methanol, one of chloroform and four of sterile dH_2_O were added to one volume of centrifuged CF, and the mixture was vortexed and centrifuged at 4500 ***g*** for 15 min. Precipitated proteins were washed with two volumes of methanol. The air‐dried pellet was resuspended in 50 μl 2 × SDS loading buffer and prepared for SDS‐PAGE as described earlier.

To immunoprecipitate effector mRFP fusion proteins from *P. infestans* CF, total proteins in 10 ml CF were precipitated using acetone precipitation (Fic *et al*., 2010). Briefly, four volumes of chilled acetone were added to one volume of CF, and the mixture was vortexed and incubated at −20°C overnight. The mixture was centrifuged at 4500 ***g*** for 10 min at 4°C. The pellet was air‐dried and dissolved in 1 ml GTEN buffer (10% glycerol, 25 mM Tris–HCl pH = 7.5, 1 mM ethylenediaminetetraacetic acid (EDTA), 150 mM NaCl) with 10 mM dithiothreitol (DTT), protease inhibitor cocktail (PIC), 1 mM phenylmethylsulfonyl fluoride (PMSF) and 0.2% Nonidet P‐40 added, and centrifuged at 13 000 ***g*** for 10 min at 4°C. The supernatant was mixed with 40 μl RFP‐Trap® magnetic agarose beads (Chromotek, Manchester, UK) and rotated at 4°C overnight. Beads were washed five times using GTEN buffer with PIC and 1 mM PMSF added. The washed beads were resuspended in 50 μl 2× SDS loading buffer and heated at 95°C for 10 min. The supernatant was magnetically separated from the beads and taken for SDS‐PAGE.

The extraction and immunoprecipitation of effector mRFP fusion proteins from *N. benthamiana* leaves infiltrated with *Agrobacterium* containing overexpression vectors were carried out as described by Wang *et al*. (2017) and He *et al*. (2018).

### SDS‐PAGE and western blot

Samples were loaded onto 12% self‐cast gels; gel electrophoresis, membrane blocking and washing steps were performed as described by McLellan *et al*. (2013). Amersham™ Protran™ 0.45 μm NC nitrocellulose blotting membrane was used. Primary RFP antibody (α RFP; Chromotek) and histone H3 antibody (α H3; Abcam, Cambridge, UK) were used at 1: 5000 dilutions. Secondary anti‐rat immunoglobulin G (IgG) horseradish peroxidase (HRP) antibody (Abcam) and anti‐rabbit IgG HRP antibody (Sigma‐Aldrich) were used at 1 : 5000 dilutions. Protein bands on membranes were visualised using high‐performance chemiluminescence film (Amersham™ Hyperfilm™ ECL) and Pierce™ or SuperSignal™ ECL western blotting substrate (Thermo Fisher Scientific, Waltham, MA, USA).

### Confocal microscopy

*Nicotiana benthamiana* leaves infected by *P. infestans* (4–6 d post inoculation) or infiltrated with *Agrobacterium* containing overexpression vectors (2 d post infiltration) were infiltrated with dH_2_O and then mounted on slides. A Nikon A1R confocal microscope was used for imaging. RFP was imaged with 561 nm excitation, and emissions were collected between 570 and 620 nm. GFP and chloroplast autofluorescence were imaged with 488 nm excitation, and emissions were collected between 500–530 nm and 663–738 nm, respectively. The pinhole was set to 1.2 Airy units for the longest wavelength fluorophore of any combination. All images were processed and analysed using the fiji software (Schindelin *et al*., 2012).

### Liquid chromatography–tandem mass spectrometry (LC‐MS/MS)

Immunoprecipitated samples from the CF of *P. infestans* transformants were analysed by SDS‐PAGE. Protein bands were visualised by InstantBlue Coomassie Protein Stain (Abcam) staining. Bands of interest were excised, destained and digested overnight (16 h) with trypsin, chymotrypsin or lys C (MS Grade, Pierce; Thermo Fisher Scientific). Peptides generated by the digestion were extracted from the gel and dried in a SpeedVac (Thermo Fisher Scientific). Dried peptides were resuspended with 40 μl 1% formic acid, centrifuged and transferred to HPLC vials for analysis using Ultimate™ 3000 RSLCnano system (Thermo Fisher Scientific) coupled to a Q Exactive™ Plus Mass Spectrometer (Thermo Fisher Scientific). Mass spectrometry analysis was carried out at the ‘FingerPrints’ Proteomics Facility, School of Life Sciences, University of Dundee. 15 μl samples was injected, and peptides were initially trapped on an Acclaim™ PepMap™ 100 (C18, 100 μM × 2 cm), then separated on an Easy‐Spray™ PepMap RSLC C18 column (75 μM × 50 cm) (Thermo Fisher Scientific) and transferred to the mass spectrometer via an Easy‐Spray source with temperature set at 50°C. In data‐dependent acquisition mode, full MS1 scans were performed at 70000 resolution followed by 15 sequential‐dependent MS2 scans where the top 15 ions were selected for collision‐induced dissociation (CID, normalised collision energy NCE = 35.0) and analysis in the Ion Trap with an MSn AGC target of 5000. An isolation window of 2.0 *m/z* units around the precursor was used, and selected ions were then dynamically excluded from further analysis. The Mascot data software (v.2.6) was used for data analysis.

## Results

### Cleavage of the RXLR motif in RXLR‐only effectors

**Fig. 1:**
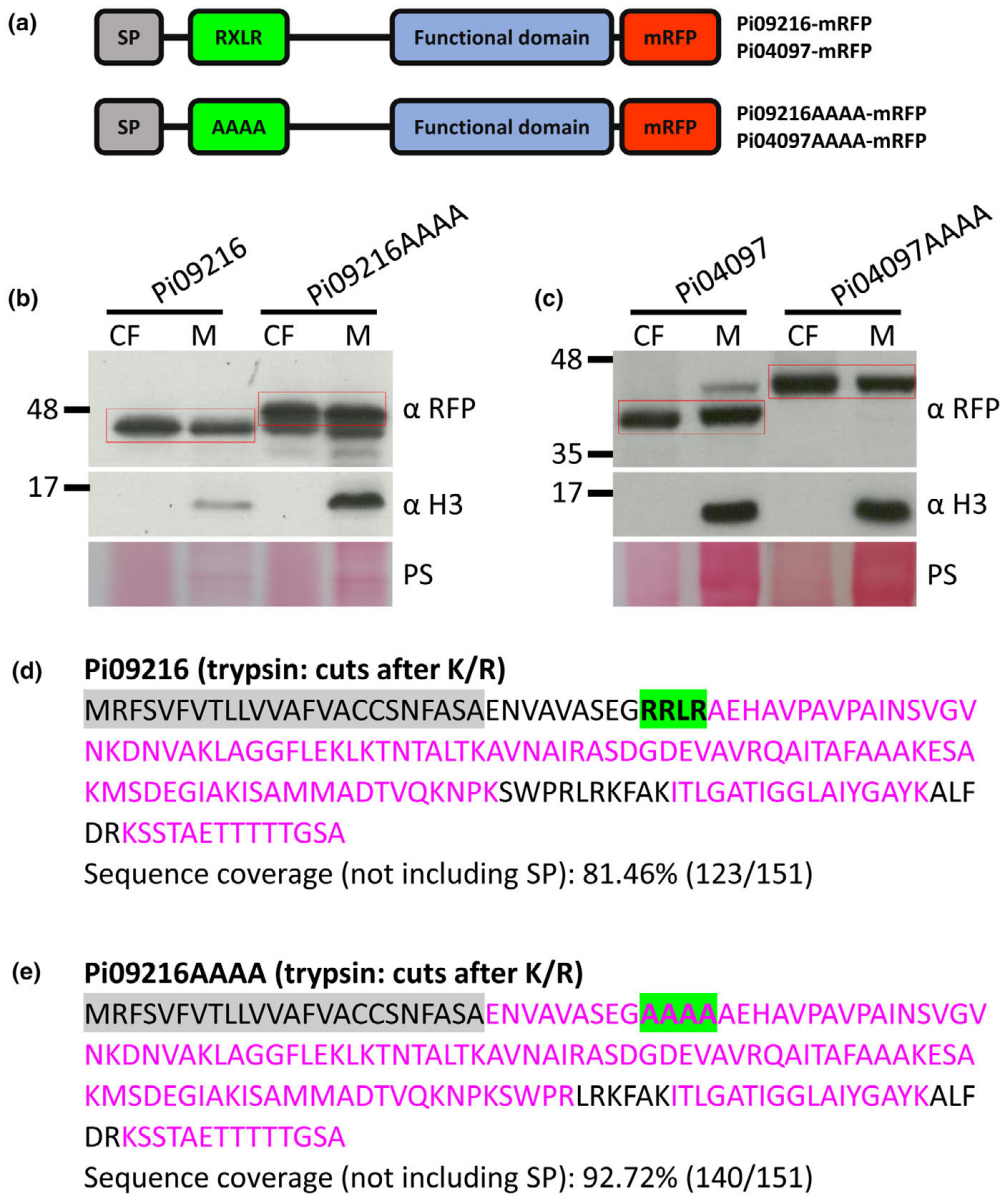
Cleavage of the RXLR motif in RXLR‐only effectors. (a) Schematic representation of Pi09216 and Pi04097 wild‐type (WT) and RXLR mutant (AAAA) monomeric red fluorescent protein (mRFP) fusions. SP, signal peptide. Pi09216 (b) and Pi04097 (c) WT and AAAA mRFP fusions (enclosed by red rectangles) were detected in the culture filtrate (CF) and mycelium (M) using RFP antibody (αRFP). *Phytophthora infestans* histone H3 was detected using histone antibody (α H3) only in the M which indicates that the CF was not detectably contaminated by cellular material. Markers (kDa) are indicated on the left and protein loading is indicated by Ponceau stain (PS). Distribution of the LC‐MS/MS identified peptides within the sequences of Pi09216 WT (d) and AAAA (e). Peptides were generated by trypsin in‐gel digestion. Identified peptides are marked with magenta, amino acids not identified in peptides are marked with black, predicted SP is shaded with grey, and the RXLR motif and its alanine replacement (AAAA) are shaded with green. K, lysine; R, arginine.

Cleavage at the RXLR motif of PiAVR3a detected in the culture filtrate (CF) of axenically grown *P. infestans* was reported previously (Wawra *et al*., 2017). To investigate whether such cleavage was widespread, we selected two effectors that contain the RXLR motif but lack the EER motif, Pi09216 and Pi04097, each of which enhances *P. infestans* colonisation when transiently expressed in *N. benthamiana* (Wang *et al*., 2019). Pi04097 is an AvrBlb2 family member and is recognised by the potato NLR RpiBlb2 (Oh *et al*., 2009). Constructs expressing either the WT versions of each effector or versions in which the RXLR was mutated to alanines (AAAA), each fused to mRFP at the C‐terminus, were transformed into *P. infestans* (Fig. 1a). Transformants expressing the mutant forms, Pi09216AAAA or Pi04097AAAA, generated proteins of a size consistent with cleavage after the signal peptide (SP). These effector forms were detected in both the CF and mycelium samples. Histone H3 was used as a control of cellular proteins and revealed a band of the expected size in mycelium samples. By contrast, histone H3 was not detected in CF samples, demonstrating that there was no cell lysis to release cellular proteins into the CF. The detection of WT and mutant effector forms in the CF was thus due to active secretion rather than cell lysis (Fig. 1b,c). Transformants expressing the WT versions of Pi09216‐mRFP and Pi04097‐mRFP generated smaller fusion proteins consistent with cleavage at the RXLR motif (red boxes in Fig. 1b,c). The smaller fusion proteins were detected in mycelium samples, suggesting that processing of the effectors occurred before secretion. These smaller fusion proteins were also evident in transformants expressing mutant Pi09216AAAA‐mRFP samples, perhaps indicating that cleavage was reduced rather than fully prevented by the mutation. In the case of WT Pi04097‐mRFP, a faint fusion protein of a size predicted to be the SP cleavage product was detected only in the mycelium sample (Fig. 1c). Both the WT and mutant forms of Pi09216‐mRFP and Pi04097‐mRFP were evident around the outside of GFP‐labelled haustoria in fluorescence intensity plots, consistent with them being secreted from haustoria during infection, indicating that mutation of the RXLR motif does not prevent this (Fig. S1a,b). To test whether similar processing of the WT effectors occurred during interactions with host cells, transformants expressing the WT and mutant mRFP fusions of Pi09216 were inoculated onto *N. benthamiana* leaves and the WT and mutant Pi09216‐mRFP fusion proteins were immunoprecipitated. The WT and mutant fusion proteins were similar sizes during infection and growth in culture medium (Fig. S2a), indicating that similar processing occurs in each case.

WT and mutant forms of Pi09216‐mRFP were immunoprecipitated from CF following growth of *P. infestans* in axenic culture and LC‐MS/MS was conducted from replicate experimental samples. Whereas peptides were only detected representing sequences from the RXLR motif to the C‐terminus of the WT Pi09216 (Fig. 1d; Table S2a), consistent with cleavage at the motif, peptides from the predicted SP cleavage site, spanning the AAAA region of mutated Pi09216, were reproducibly detected (Fig. 1e; Table S2b), confirming that uncleaved protein was secreted by the transformant expressing the mutated effector.

### Cleavage of effectors with both RXLR and EER motifs

**Fig. 2:**
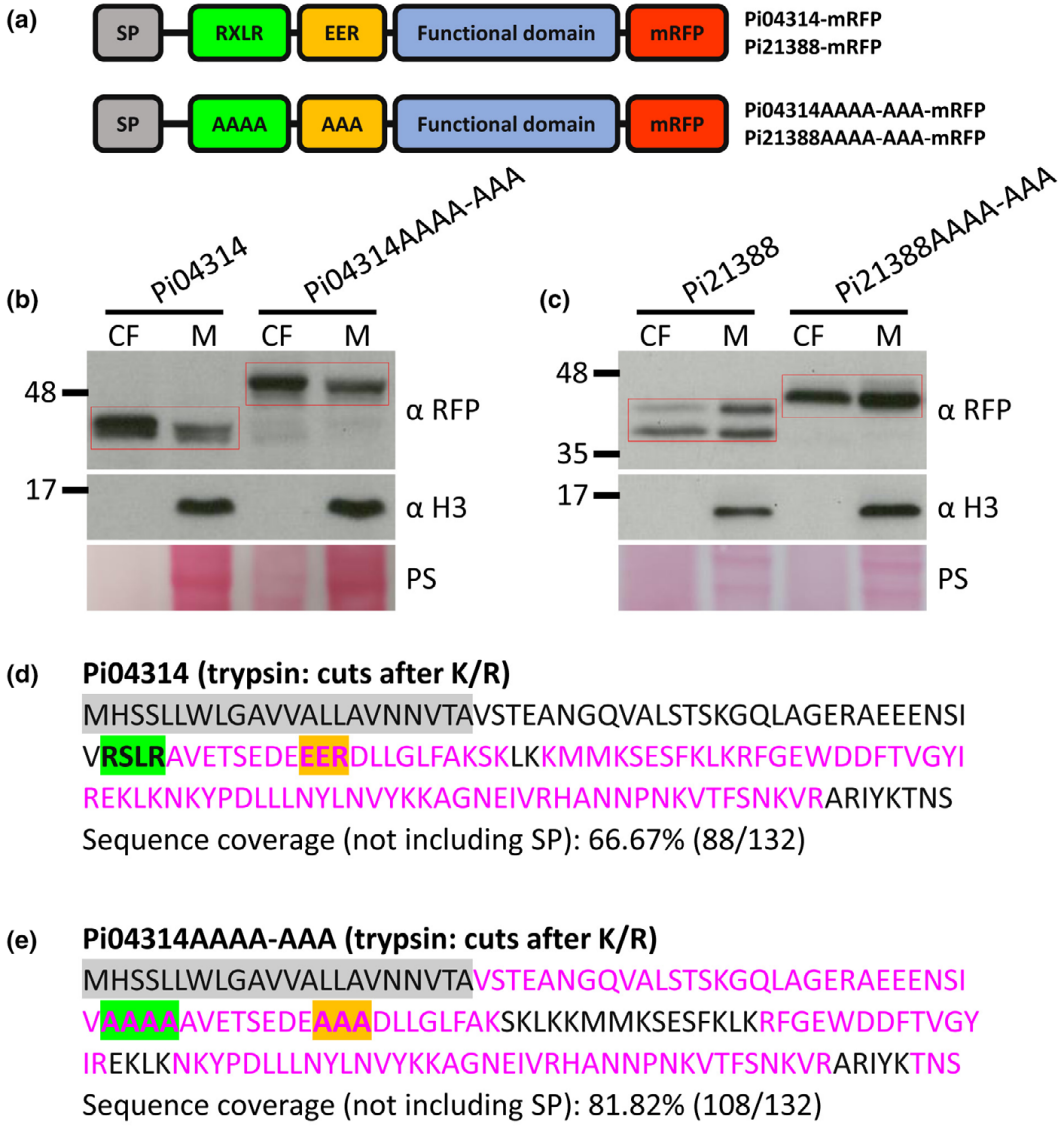
Cleavage of the RXLR motif in RXLR‐EER effectors. (a) Schematic representation of Pi04314 and Pi21388 WT and RXLR‐EER mutant (AAAA‐AAA) mRFP fusions. Pi04314 (b) and Pi21388 (c) WT and AAAA‐AAA mRFP fusions (enclosed by red rectangles) were detected in the culture filtrate (CF) and mycelium (M) using αRFP. *Phytophthora infestans* histone H3 was detected using α H3 only in the M which indicates that the CF was not detectably contaminated by cellular material. Markers (kDa) are indicated on the left and protein loading is indicated by Ponceau stain (PS). Distribution of the LC‐MS/MS identified peptides within the sequences of Pi04314 WT (d) and AAAA‐AAA (e). Peptides were generated by trypsin in‐gel digestion. Identified peptides are marked with magenta, amino acids not identified in peptides are marked with black, predicted SP is shaded with grey, the RXLR motif and its alanine replacement (AAAA) are shaded with green, and the EER motif and its alanine replacement (AAA) are shaded with gold. α H3, histone H3 antibody; α RFP, RFP antibody; CF, culture filtrate; K, lysine; M, mycelium; mRFP, monomeric red fluorescent protein; R, arginine; PS, ponceau stain; SP, signal peptide.

To study in detail the potential for proteolytic processing of effectors containing both the RXLR and EER motifs, two well‐characterised effectors were selected: Pi04314, which is secreted from haustoria (Wang *et al*., 2017) and interacts with host PP1c isoforms in the plant nucleus (Boevink *et al*., 2016; Bentham *et al*., 2023); and Pi21388 (IPIO1/Avrblb1), which is recognised by the potato resistance RpiBlb1 (Vleeshouwers *et al*., 2008). Initially, WT versions of each effector, and versions in which both RXLR and EER motifs were mutated (AAAA‐AAA), were expressed in *P. infestans* as mRFP fusions (Fig. 2a). The AAAA‐AAA mutants generated fusion proteins of a size expected following cleavage at the SP. These were evident in the mycelium samples and were secreted into CF (Fig. 2b,c). By contrast, smaller fusion proteins were observed for both WT Pi04314‐mRFP and Pi21388‐mRFP (Fig. 2b,c). This pattern was also evident for WT and AAAA‐AAA mutant versions of RXLR‐EER effector Pi22926 (Fig. S3), which has been shown previously to be secreted at haustoria and translocated into host cells (Wang *et al*., 2018). Fluorescence intensity plots indicated that both the WT and AAAA‐AAA mutant forms of Pi04314, Pi21388 and Pi22926 were secreted from haustoria during infection, indicating that mutation of the RXLR and EER motifs does not prevent this (Fig. S4). To test whether similar processing of the WT effectors occurred during interactions with host cells, transformants expressing the WT and mutant mRFP fusions of Pi04314 were inoculated onto *N. benthamiana* leaves and the Pi04314‐mRFP fusion proteins were immunoprecipitated. The WT and mutant fusion proteins were similar sizes during infection and growth in culture medium (Fig. S2b), indicating that similar processing likely occurs in each case.

WT and mutant Pi04314‐mRFP fusion proteins were immunoprecipitated from CF following growth of *P. infestans* in axenic culture and LC‐MS/MS was conducted from replicate experimental samples. Peptides were only detected from after the RXLR motif to the C‐terminus of the WT Pi04314 (Fig. 2d; Table S2c), consistent with cleavage at the motif. By contrast, peptides spanning the AAAA and AAA regions of mutated Pi04314 were reproducibly detected (Fig. 2e; Table S2d), consistent with a much‐reduced cleavage in the AAAA‐AAA mutant effector.

### Evidence of cleavage at both RXLR and EER motifs

**Fig. 3:**
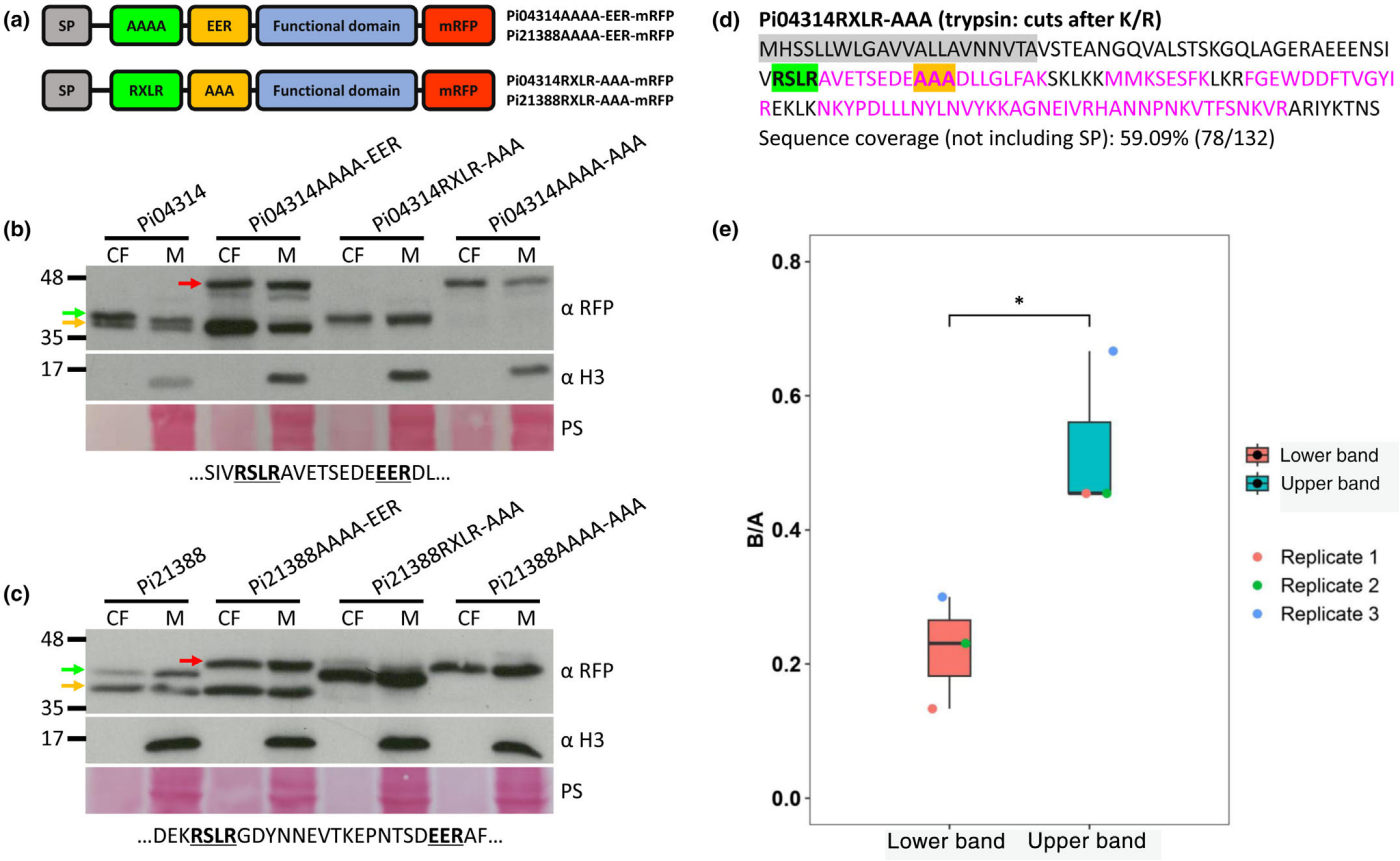
Evidence of cleavage at both RXLR and EER motifs. (a) Schematic representation of Pi04314 and Pi21388 RXLR mutant (AAAA‐EER) and EER mutant (RXLR‐AAA) mRFP fusions. Pi04314 (b) and Pi21388 (c) wild‐type (WT), AAAA‐EER, RXLR‐AAA and AAAA‐AAA mRFP fusions were detected in the culture filtrate (CF) and mycelium (M) using αRFP. *Phytophthora infestans* histone H3 was detected using α H3 only in the M, which indicates that the CF was not detectably contaminated by cellular material. Gold arrows indicate fusions cleaved at the EER motif, green arrows indicate fusions cleaved at the RXLR motif, and red arrows indicate fusions cleaved after the SP. Markers (kDa) are indicated on the left and protein loading is indicated by Ponceau stain (PS). Partial effector sequences containing the RXLR and EER motifs are shown below the PS. Immunoblots are representative of three independent biological replicates. (d) Distribution of the LC‐MS/MS identified peptides within the sequence of Pi04314RXLR‐AAA. Peptides were generated by trypsin in‐gel digestion. Identified peptides are marked with magenta, amino acids not identified in peptides are marked with black, predicted SP is shaded with grey, the RXLR motif is shaded with green, and the EER motif alanine replacement (AAA) is shaded with gold. (e) Graph shows the ratio of the number of times peptides immediately before (AEEENSIVAAAAAVETSEDEEER and GQLAGERAEEENSIVAAAAAVETSEDEEER, B) and after (DLLGLFAK and DLLGLFAKSK, A) the EER motif were detected. A two‐tailed *t*‐test with unequal variances indicates a statistically significant difference between the ratios of the lower and upper bands of Pi04314AAAA‐EER (*, *P* < 0.05, three replicates). α H3, histone H3 antibody; α RFP, RFP antibody; CF, culture filtrate; K, lysine; M, mycelium; mRFP, monomeric red fluorescent protein; R, arginine; PS, ponceau stain; SP, signal peptide.

We noted that WT Pi21388‐mRFP transformants revealed two protein bands in both mycelium and CF samples (Fig. 2c). Close inspection revealed that two fusion proteins were apparent also for WT Pi04314‐mRFP (Fig. 2b) but these were closer in size. The differences in size between the fusion protein pairs produced by the transformants expressing Pi21388‐mRFP and Pi04314‐mRFP may reflect distance between the RXLR and EER motifs; 15 amino acids in the case of Pi21388 and eight amino acids in the case of Pi04314‐mRFP (Fig. 3). Two fusion protein bands were not initially evident in the case of WT Pi22926‐mRFP (Fig. S3). This may reflect that only three amino acids separate RXLR and EER motifs in Pi22926‐mRFP (Fig. S5). To test the hypothesis that each motif was a cleavage site, we mutated either the RXLR (AAAA‐EER) or the EER (RXLR‐AAA) motif independently for each effector (Fig. 3a). As observed previously (Figs 2, S3), the double mutants (AAAA‐AAA) of Pi04314‐mRFP, Pi21388‐mRFP and Pi22926‐mRFP generated protein bands of a size consistent with cleavage at the SP in both mycelium and CF samples. Moreover, WT fusion proteins for each effector revealed smaller bands consistent with cleavage at the conserved motifs (Figs 3b,c, S5). Effector forms in which the RXLR (AAAA‐EER) was mutated generated two fusion products; one with predicted cleavage at the SP and one consistent with cleavage at the EER motif (Figs 3b,c, S5). Effector forms in which the EER (RXLR‐AAA) motif was mutated generated a dominant fusion product of a size predicted following cleavage at the RXLR motif (Figs 3b,c, S5). Each of the single‐motif mutant transformants expressed mRFP fusion proteins that were secreted at haustoria during infection (Fig. S6).

To further confirm that the size difference between the effector RXLR and EER mutant mRFP fusions resulted from the different cleavage at the SP, RXLR or EER motifs, secreted Pi04314RXLR‐AAA and Pi04314AAAA‐EER mRFP fusions were immunoprecipitated from CF and analysed by LC‐MS/MS. Peptides generated by trypsin in‐gel digestion were only detected from the RXLR motif to the C‐terminus of Pi04314RXLR‐AAA (Fig. 3d; Table S2e), which suggests that the fusion was cleaved at the RXLR motif. As two obvious bands were observed in the CF of Pi04314AAAA‐EER (Figs 3b, S7a,b), we excised and analysed them separately. For the upper band, peptides generated by trypsin in‐gel digestion were detected immediately after the predicted SP to the C‐terminus (Fig. S7c; Table S2f), which suggests that this band is the product from cleavage after the SP. Similar sequence coverage as the upper band was observed with the lower band (Fig. S7d; Table S2g). Considering the close distance between the upper (≈ 42 kDa) and lower (≈ 36 kDa) bands and the inefficiency of SDS‐PAGE to separate the two bands completely (Fig. S7b), it is likely that the detected peptides before the EER motif in the lower band were due to contamination from the upper band, or due to breakdown products. To mitigate for this, we calculated the ratio of the number of times peptides immediately before (AEEENSIVAAAAAVETSEDEEER and GQLAGERAEEENSIVAAAAAVETSEDEEER) and after (DLLGLFAK and DLLGLFAKSK) the EER motif were detected in three replicate experiments. Critically, the upper band has a higher ratio compared with the lower band, and there is a statistically significant difference between them across replicates (Fig. 3e; Table S2f,g).

**Fig. 4:**
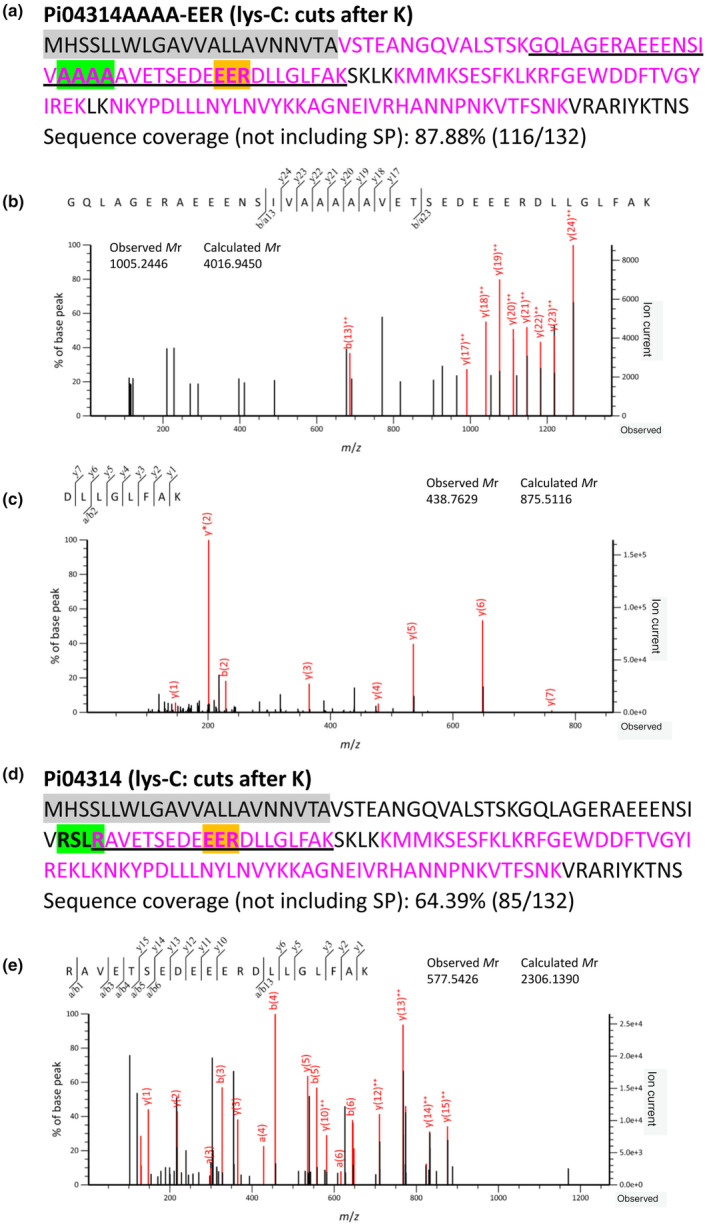
Cleavage of RXLR and EER motifs occurs after the leucine (L) and arginine (R), respectively. Distribution of the LC‐MS/MS identified peptides within the sequences of Pi04314AAAA‐EER (a) and wild‐type (WT) (d). Peptides were generated by lys‐C in‐gel digestion. Identified peptides are marked with magenta, amino acids not identified in peptides are marked with black, predicted SP is shaded with grey, the RXLR motif and its alanine replacement (AAAA) are shaded with green, and the EER motif is shaded with gold. (b) MS/MS fragmentation of the identified peptide GQLAGERAEEENSIVAAAAAVETSEDEEERDLLGLFAK (underlined in black in a) containing the alanine replacement of the RXLR motif and the EER motif. (c) MS/MS fragmentation of the identified peptide DLLGLFAK (underlined in black in a) starting immediately after the R of the EER motif. (e) MS/MS fragmentation of the identified peptide RAVETSEDEEERDLLGLFAK (underlined in black in d) starting immediately after the L of the RXLR motif. K, lysine; SP, signal peptide.

Since we were unable to separate the upper and lower bands of Pi04314AAAA‐EER completely, we excised them together and digested with lys‐C, which cuts only after lysine (K), instead of trypsin, which cuts after arginine (R) or K. Peptide coverage from immediately after the predicted SP to the C‐terminus was observed (Fig. 4a), which is consistent with the trypsin digestion. A long peptide containing the alanine replacement of the RXLR motif, followed by the EER motif (GQLAGERAEEENSIVAAAAAVETSEDEEERDLLGLFAK), was detected multiple times (Fig. 4b; Table S2h), which further confirms that mutation of the RXLR motif to AAAA prevented cleavage at this site and the upper band resulted from the cleavage after the SP. Notably, multiple copies of a peptide from immediately after the EER motif (DLLGLFAK) were detected (Fig. 4c; Table S2h), which indicates that the lower band resulted from the endogenous cleavage after the R of the EER motif, since lys‐C does not cut after R.

### The RXLR motif is cleaved after the leucine (L)

In the LC‐MS/MS analysis of Pi04314‐mRFP fusion protein peptides were detected from beyond the second R in the RXLR motif, which is consistent with the previous report about the cleavage of Avr3a (Wawra *et al*., 2017). However, the detected peptides here and in the previous report were generated by in‐gel digestion using trypsin (which cuts at the carboxyl side of R and K), which means the cleavage could be performed either by protease(s) of *P. infestans* before secretion or by trypsin during in‐gel digestion. To further examine the precise site of cleavage at the RXLR motif, we first used the protease chymotrypsin (which cuts at the carboxyl side of F, L, W and Y) for in‐gel digestion to test whether a peptide starting immediately after the second R could still be detected. Peptides from Pi04314‐mRFP digested with chymotrypsin represented sequences after the RXLR motif, which is consistent with trypsin digestion (Fig. S8a). However, no peptides starting immediately after the second R were detected, whereas peptides starting immediately after the L (RAVETSEDEEERDLL) were detected (Fig. S8b; Table S2i). This suggests that cleavage does not occur after the second R of the RXLR motif.

Since chymotrypsin cuts after L, we used the protease lys‐C, which cuts at the carboxyl side of K, to account for the possibility that cleavage after the L occurred during in‐gel digestion. Peptide coverage of Pi04314‐mRFP generated by lys‐C digestion was from the RXLR motif to the C‐terminus (Fig. 4d), which is consistent with the trypsin and chymotrypsin digestions. Similarly, multiple peptides starting immediately after the L (RAVETSEDEEERDLLGLFAK) were detected (Fig. 4e; Table S2j), which confirms that cleavage in *P. infestans* at the RXLR motif occurs between the L and the second R. Consistent with the importance of this site for cleavage, mutation of just the L and second R to alanines (RxAA) prevented cleavage at the RXLR motif, similar to the AAAA mutant (Fig. S8c). In addition, multiple copies of a peptide starting immediately after the EER motif (DLLGLFAK) were detected, which supports that endogenous cleavage also occurs after the R of the EER motif (Table S2j).

Transformants expressing two further RFP‐tagged RXLR‐EER effectors were available in the laboratory: Pi04145 (Wang *et al*., 2019) and Pi09160, which was detected in proteomics in endosomes isolated from infected plants (Wang *et al*., 2023a). Western blotting of M and CF from cultures of these transformants showed bands consistent with cleavage at the RXLR and EER motifs (Fig. S9a,b). As with other such transformants, the effector fusions were secreted at haustoria during infection (Fig. S9c,d). These observations support the hypothesis that cleavage at RXLR and EER motifs is a general rule.

### Efficient cleavage of RXLR and EER motifs does not occur during secretion from plant cells

**Fig. 5:**
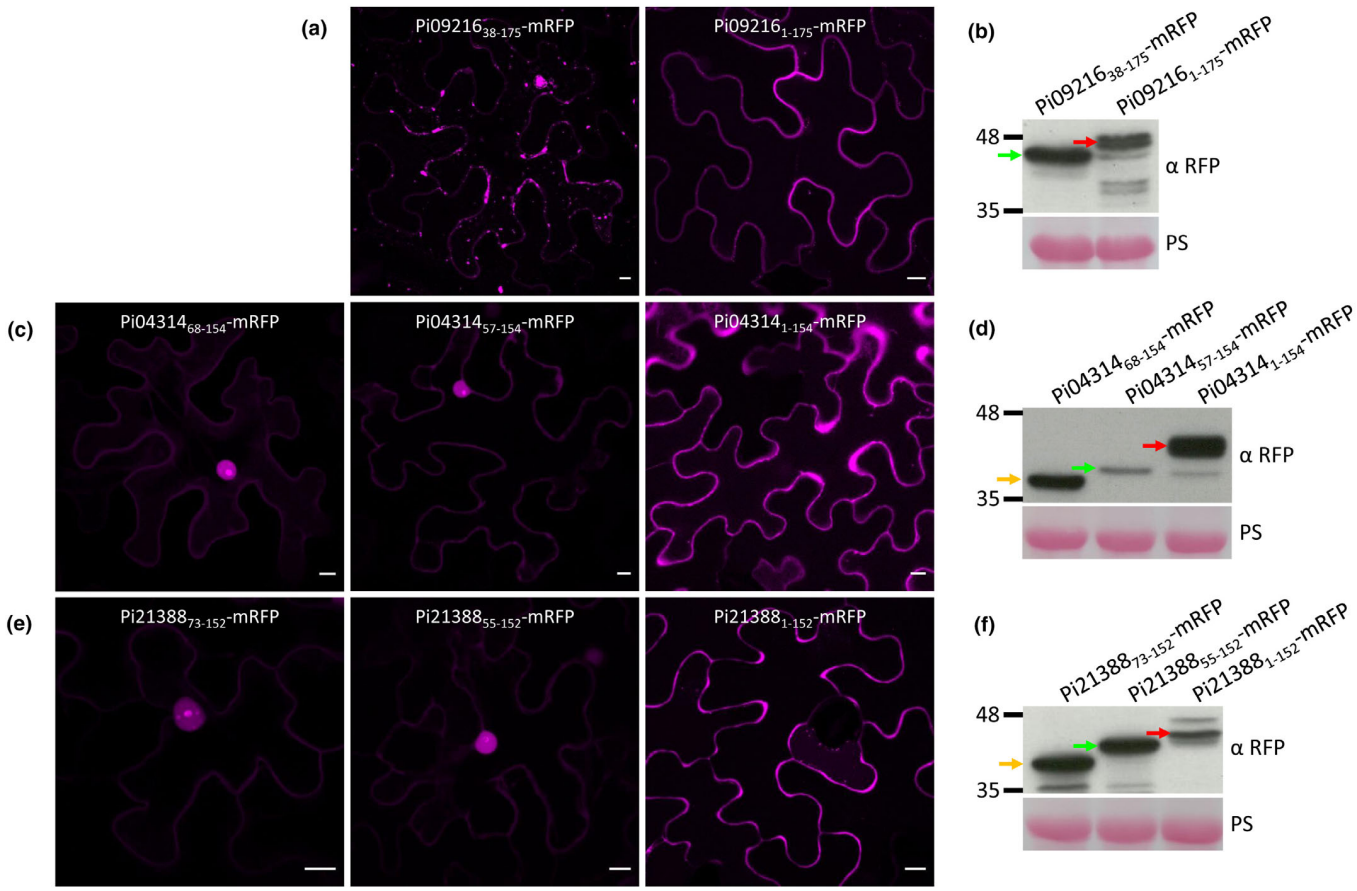
Efficient cleavage of RXLR and EER motifs does not occur during secretion from the plant. Confocal images of *Agrobacterium*‐mediated transient overexpression of Pi09216_38–175_‐mRFP (a, left), Pi09216_1–175_‐mRFP (a, right), Pi04314_68–154_‐mRFP (c, left), Pi04314_57–154_‐mRFP (c, middle), Pi04314_1–154_‐mRFP (c, right), Pi21388_73–152_‐mRFP (e, left), Pi21388_55–152_‐mRFP (e, middle) and Pi21388_1–152_‐mRFP (e, right) in *Nicotiana benthamiana* cells. Images were taken 2 d post infiltration. Bars, 10 μm. Expression of Pi09216_38–175_‐mRFP and Pi09216_1–175_‐mRFP (b), Pi04314_68–154_‐mRFP, Pi04314_57–154_‐mRFP and Pi04314_1–154_‐mRFP (d), and Pi21388_73–152_‐mRFP, Pi21388_55–152_‐mRFP and Pi21388_1–152_‐mRFP (f) fusions was confirmed by immunoblotting using α RFP. Markers (kDa) are indicated on the left and protein loading is indicated by Ponceau stain (PS). Red, green and gold arrows indicate effector mRFP fusions starting after the SP, RXLR motif and EER motif, respectively. α RFP, RFP antibody; mRFP, monomeric red fluorescent protein; PS, ponceau stain.

To test whether RXLR‐only and RXLR‐EER effectors of *P. infestans* would be similarly proteolytically processed during secretion from other eukaryotic cells, we expressed full‐length (including SP) versions of Pi09216‐mRFP (RXLR‐only), Pi04314‐mRFP and Pi21388‐mRFP (RXLR‐EER) in *N. benthamiana* cells. As controls, we also expressed nonsecreted truncated versions from the end of the RXLR to the C‐terminus (Pi09216_38–175_‐mRFP, Pi04314_57–154_‐mRFP and Pi21388_55–152_‐mRFP) and from the end of the EER to the C‐terminus (Pi04314_68–154_‐mRFP and Pi21388_73–152_‐mRFP) in *N. benthamiana*. The truncated versions were each of an expected size and revealed subcellular localisations observed previously (Fig. 5); Pi09216‐mRFP at small puncta shown previously to include mitochondria (Wang *et al*., 2019) and Pi04314‐mRFP and Pi21388‐mRFP localised primarily in host nuclei and nucleoli (Boevink *et al*., 2016; Wang *et al*., 2019, 2023a). By contrast, the full‐length effector fusions with SP were each localised at the cell periphery, consistent with them being secreted by the host cells (Fig. 5a,c,e). Interestingly, the full‐length Pi09216‐mRFP was primarily of a size predicted from processing at the SP and was only very weakly cleaved to generate a fusion protein of the size expected from RXLR cleavage (Fig. 5b). The full‐length versions of Pi04314‐mRFP and Pi21388‐mRFP again each showed fusion proteins primarily processed after the SP, faint bands of a size consistent with weak cleavage at the RXLR motif, and no evidence of processing at the EER motif (Fig. 5d,f).

### Cleavage of the RXLR motif is related to its position

**Fig. 6:**
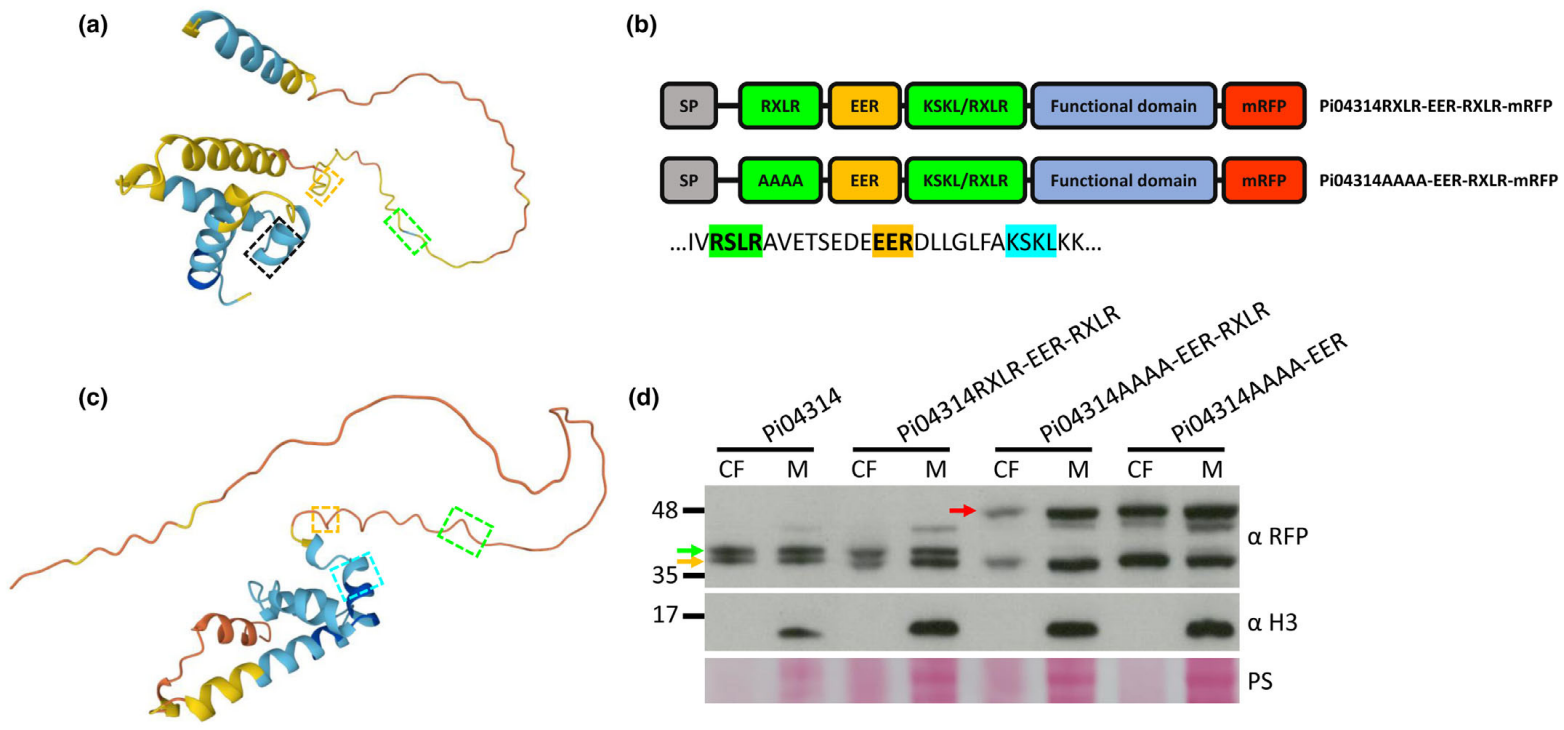
Cleavage of the RXLR motif is related to its position. Predicted structures of Pi21388 (a) and Pi04314 (c) obtained from the AlphaFold Protein Structure Database (https://alphafold.ebi.ac.uk/). RXLR motifs in the N‐terminus are enclosed by green dashed rectangles, EER motifs are enclosed by gold dashed rectangles, another RXLR motif of Pi21388 in the C‐terminus is enclosed by the black dashed rectangle, and the KSKL amino acids of Pi04314 are enclosed by the cyan dashed rectangle. (b) Schematic representation of Pi04314RXLR‐EER‐RXLR and Pi04314AAAA‐EER‐RXLR mRFP fusions. Partial Pi04314 sequence containing the RXLR (shaded with green), EER (shaded with gold) motifs and the KSKL amino acids (shaded with cyan) is shown below. (d) Pi04314 WT, RXLR‐EER‐RXLR, AAAA‐EER‐RXLR and AAAA‐EER mRFP fusions were detected in the culture filtrate (CF) and mycelium (M) using α RFP. *Phytophthora infestans* histone H3 was detected using α H3 only in the M which indicates that the CF was not detectably contaminated by cellular material. Gold arrow indicates fusions cleaved at the EER motif, green arrow indicates fusions cleaved at the RXLR motif, and red arrow indicates fusions cleaved after the SP. Markers (kDa) are indicated on the left and protein loading is indicated by Ponceau stain (PS). Immunoblot is representative of three independent biological replicates (Supporting Information Fig. S10). α H3, histone H3 antibody; α RFP, RFP antibody; CF, culture filtrate; M, mycelium; mRFP, monomeric red fluorescent protein; PS, ponceau stain; SP, signal peptide.

There are two RXLR motifs within the sequence of effector Pi21388: One is in the predicted unstructured region of the N‐terminus and the other is located within a short alpha helix in the C‐terminal domain (Fig. 6a). According to immunoblots detecting WT Pi21388‐mRFP in M and CF samples (Figs 2c, 3c), only the N‐terminal RXLR motif was cleaved, which suggests that cleavage of RXLR motif could be related to its location within the sequence. To test this hypothesis, an extra RXLR motif was introduced into the sequence of WT Pi04314 and RXLR mutant Pi04314AAAA‐EER by mutating the original amino acid residues KSKL, which are seven residues downstream of the EER motif and located within a predicted alpha helix, to RSLR (Fig. 6b,c). Transformants overexpressing Pi04314RXLR‐EER‐RXLR‐mRFP or Pi04314AAAA‐EER‐RXLR‐mRFP were generated and the secretion of these mRFP fusions at haustoria during infection was confirmed by confocal microscopy (Fig. S10a). The CF and M of these transformants grown *in vitro* were used for protein precipitation and extraction, respectively, and samples were analysed by immunoblotting along with samples from the Pi04314 WT and AAAA‐EER transformants. The number and size of Pi04314RXLR‐EER‐RXLR‐mRFP protein bands are the same as WT Pi04314‐mRFP (Fig. 6d), which suggests that the introduced RXLR motif is not cleaved. Moreover, the number and size of Pi04314AAAA‐EER‐RXLR‐mRFP protein bands is also the same as Pi04314 AAAA‐EER‐mRFP (Fig. 6d), which indicates that the introduced RXLR motif is not proteolytically cleaved. Similar results were observed in an additional two replicates (Fig. S10b). Together, we concluded that cleavage of the RXLR motif is related to its position.

## Discussion

**Fig. 7:**
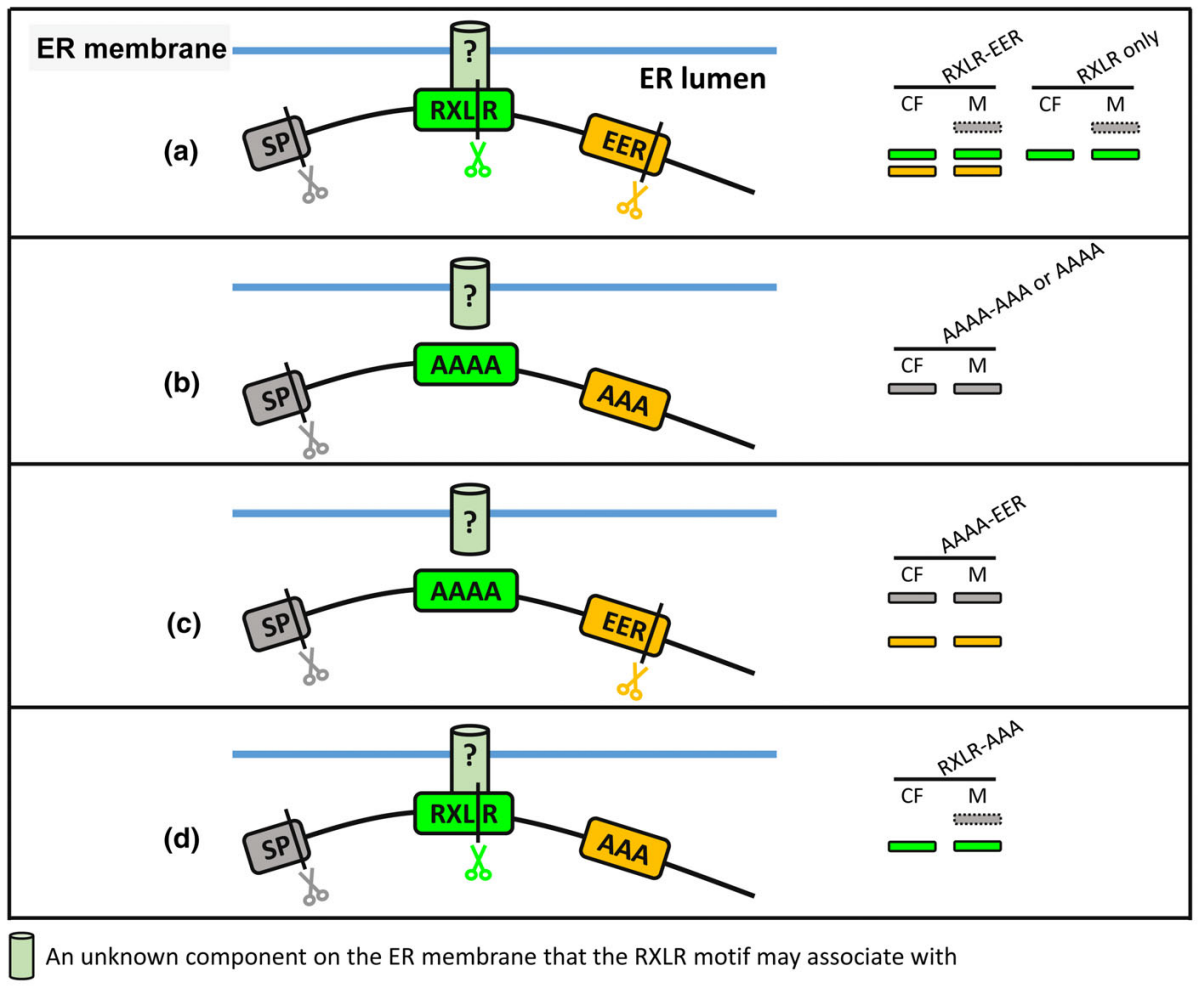
A model for RXLR(−EER) effector cleavage. SP directs effector into the endoplasmic reticulum (ER), where SP is cleaved. The RXLR motif is associated with an unknown component (indicated as light green cylinders), potentially at the ER membrane (indicated as blue straight lines). (a) Wild‐type (WT) RXLR‐EER effectors are cleaved at the RXLR and EER motifs, resulting in the presence of two lower bands in the CF and M (cleaved at the RXLR and EER motifs, represented by green and gold rectangles), or one lower band for RXLR‐only effectors. Some effectors are incompletely cleaved resulting in the presence of a higher band (cleaved after the SP, represented by the grey‐dashed rectangles), which is retained in the M due to the association with an unknown component. (b) When both the RXLR and EER motifs are mutated, the association between the RXLR and the unknown component and the cleavage events are abolished, resulting in only one higher band in both the CF and M (cleaved after the SP, represented by grey rectangles). (c) When only mutating the RXLR motif, the association between the RXLR and the unknown component is abolished, the cleavage at the RXLR is disrupted but the cleavage at the EER still occurs, resulting in the presence of one higher band (cleaved after the SP, grey rectangles) and one lower band (cleaved at the EER, gold rectangles). (d) When only mutating the EER motif, the RXLR is still associated with the unknown component, the cleavage at the RXLR still occurs, but the cleavage at the EER is disrupted, resulting in the presence of one lower band (cleaved at the RXLR, green rectangles). Incompletely cleaved effectors (cleaved after the SP, grey dashed rectangle) are retained in the M. Black vertical lines indicate the cleavage sites of the SP, RXLR and EER cleavage. The grey scissors represent the signal peptidase that cleaves SP, the green scissors represent the protease that cleaves at the RXLR, and the gold scissors represent the protease that cleaves at the EER. CF, culture filtrate; ER, endoplasmic reticulum; M, mycelium; SP, signal peptide.

In this study it was found that the RXLR and EER motifs are two independent proteolytic cleavage sites within *P. infestans* RXLR effectors that are processed before secretion. These processing steps appears to be shared generally by different RXLR effectors. Based on the immunoblotting and proteomics analyses, a model of how RXLR effectors are processed before secretion was proposed (Fig. 7).

Proteolytic cleavage at the RXLR motif was observed in the effector Avr3a and was reported to occur after the second R (Wawra *et al*., 2017). In this study, we also observed cleavage at the RXLR motif in several RXLR effectors, thus demonstrating that this is likely a general processing step. However, by using different protein digestion enzymes for mass spectrometry analysis we found that the cleavage occurs after the L, not the second R for effector Pi04314. Whilst this needs to be confirmed for other RXLR effectors, cleavage after the L is consistent with processing of the RXLXE/D/Q motif of *P. falciparum* PEXEL effectors (Boddey *et al*., 2010; Russo *et al*., 2010; Marapana *et al*., 2018). Variations in sequence of the RXLR motif have been observed in effectors of downy mildews, including GXLR/RXLT in *Hyaloperonospora arabidopsidis* (Baxter *et al*., 2010), RXLK/Q/D in *Bremia lactucae* (Stassen *et al*., 2012), RXLK/Q/E in *Plasmopara halstedii* (Sharma *et al*., 2015) and RXLK/Q in white rusts such as *Albugo candida* and *A. laibachii* (Kemen *et al*., 2011; Links *et al*., 2011). These variations occur at the Rs of the RXLR motif but not the L, which supports the importance of this residue as a conserved component of the cleavage site. This fits with the observation that cleavage occurs immediately after this residue, rather than the variable second R in the motif. Further studies are needed to confirm the conservation of this processing site across the oomycetes.

The position of the RXLR motif, relative to the SP, has been postulated to be restricted (Battacharjee *et al*., 2006; Win *et al*., 2007; Win & Kamoun, 2008). Moreover, we observed that the cleaved RXLR motifs in Pi21388/AvrBlb1 and Pi04314 were in predicted disordered regions. Indeed, we observed that a second naturally occurring RXLR motif in Avrblb1, which was not cleaved, was located closely following the EER motif in a predicted structured region of the effector (Fig. 6). This prompted us to introduce a second RXLR motif into a similar location in Pi04314, again in a predicted structured region. This RXLR motif was also not cleaved (Fig. 6), supporting a hypothesis that the cleaved RXLR motifs are positioned in disordered regions within 30–40 amino acids of the SP. Indeed, it has been reported that the RXLR‐EER‐containing regions of the effectors generally are predicted to be disordered and this has been hypothesised to be associated with translocation (Shen *et al*., 2017). It is possible that such disordered regions facilitate efficient proteolytic processing of the effectors. Future work, following identification of the corresponding proteases, is needed to investigate this.

Cleavage at the EER motif was demonstrated for the first time in this study, but the biological significance of this processing event is unknown. Studies have shown that mutation at the EER motif disables translocation of Avr3a (Whisson *et al*., 2007), so it may be possible that EER cleavage is involved in the trafficking of RXLR effectors. Indeed, some effectors from *B. lactucae* and *H. arabidopsidis*, which are predicted to possess the conserved WY structural fold in the effector domain, appear to only possess the EER motif. They are nevertheless postulated to be translocated and recognised by host resistance (R) proteins (Bailey *et al*., 2011; Stassen *et al*., 2012; Wood *et al*., 2020). However, some RXLR effectors that only have the RXLR and no EER motif can also be translocated and recognised, such as those from the Avrblb2 family (Haas *et al*., 2009; Oh *et al*., 2009). This is consistent with a hypothesis that there may be a functional overlap between the RXLR and EER motifs in mediating RXLR effector translocation. Future work is needed to investigate whether RXLR or EER cleavage determines the unconventional pathway(s) for effector secretion (Wang *et al*., 2017, 2018) and whether the motifs play potentially redundant roles, perhaps explaining why some effectors only possess one of the motifs.

Identifying proteases that are involved in critical cellular processes is of great importance, as they can be potential targets for anti‐oomycete drug development. Wawra *et al*. (2017) tried to identify proteases that are responsible for the cleavage of Avr3a. Eleven pepsin‐like aspartic proteinases (APs) of *P. infestans* were tested as some of them are homologous to *P. falciparum* plasmepsin V (PMV), which cleaves PEXEL effectors. However, no cleavage activity was detected with these candidates (Wawra *et al*., 2017). Schoina *et al*. (2019) reported that PiAP10 and PiAP12 have weak cleavage activity towards *P. infestans* RXLR effector AVR4 *in vitro*. Further studies are needed to identify and test protease candidates. Protein cleavage usually involves transient interaction between the protease and its substrate. TurboID‐based proximity labelling (PL) has been demonstrated to be useful for the identification of weakly and transiently interacting proteins (Branon *et al*., 2018; Zhang *et al*., 2019). It could be adopted to identify potential protease candidates that are responsible for cleavage of the RXLR or EER motifs. Cleavage of RXLR effectors likely occurs in the ER, so we may be able to narrow down candidates based on (1) possession of a SP for ER entry, and (2) possession of an ER retention signal or a transmembrane domain (TMD) for ER retention. We demonstrated that plant proteases can only poorly cleave the RXLR motif and are incapable of cleaving the EER motif when full‐length effectors are secreted from *N. benthamiana* cells (Fig. 5b,d,f). This raises the potential that co‐expression of RXLR effectors and candidate *Phytophthora* proteases in the plant ER may provide an initial screen of protease candidates.

For RXLR‐only effector Pi04097 and RXLE‐EER effector Pi22926, there was a higher molecular weight band exclusively in mycelium samples but not the culture filtrate (CF) of a size consistent with cleavage only at the signal peptide (SP) (Figs 1c, S5c). This suggests that some WT effectors, following cleavage at the SP but before RXLR cleavage, were retained in the pathogen cell. One hypothesis is that SP cleavage occurs before RXLR processing, and the RXLR motif may be associated with an unknown component that anchors the effectors to the ER luminal membrane (Fig. 7). Cleavage at the RXLR motif subsequently releases effectors for secretion. Sometimes, this higher mycelium‐exclusive band can also be detected in the mycelium of Pi04314 WT (Figs 6d, S5a, S10b), but it is much fainter compared with Pi22926 and Pi04097 WT. We speculate that the intensity of this band is related to the efficiency of proteases releasing effectors from the association with the unknown retention component.

One possibility for the unknown component that the RXLR motif could associate with is phosphatidylinositol monophosphates (PIPs). Kale *et al*. (2010) reported that the RXLR motif can specifically bind to PI3P on the outer surface of plant (soybean root cell) and human (A549 cell) cell plasma membrane, and the binding mediates the entry of RXLR effectors into host and nonhost cells. Bhattacharjee *et al*. (2012) found that the binding between the RXLR motif of the *P. infestans* RXLR effector Nuk10 (PITG_15287) and PI3P on the ER luminal membrane of *P. falciparum* mediates export of the effector. However, RXLR‐PI3P binding is highly controversial (Ellis & Dodds, 2011; Yaeno *et al*., 2011; Wawra *et al*., 2012; Boddey *et al*., 2016). An alternative candidate for the unknown binding component could be the ER translocon complex. Marapana *et al*. (2018) demonstrated that the recessed SPs of *P. falciparum* PEXEL effectors could act as uncleaved signal anchors to retain the full‐length proteins on the ER membrane before the PEXEL cleavage by interacting with the special ER translocon complex that involves the protease PMV. Considering the phylogenetic relatedness between *P. infestans* and *P. falciparum*, it is possible that the RXLR motif may have a similar role as the recessed SPs of PEXEL effectors to anchor effectors on the ER membrane by interacting with a specialised ER translocon complex.

In conclusion, we reveal that both RXLR and EER motifs are sites for proteolytic cleavage. This is of significant interest as effectors possessing only the RXLR or EER motif have been observed, raising the possibility that they have a redundant or overlapping role in effector trafficking. This work thus establishes the need to determine the precise biological functions of each motif and to identify the proteases that cleave them. Such knowledge would provide biochemical or genetic targets for control of multiple diseases that threaten food, energy and environmental security.

## Competing interests

None declared.

## Author contributions

LX, SCW, PCB and PRJB designed the work; LX, SW, HW, WW and LW performed the research; LX, SCW, PCB and PRJB analysed and interpreted data; LX prepared the figures; SCW, PCB and PRJB secured the funding and supervised the work; LX and PRJB wrote the manuscript with input from all co‐authors.

## Supporting information


**Fig. S1** Secretion of RXLR‐only effector wild‐type (WT) and RXLR mutant (AAAA) *in planta*.
**Fig. S2** Cleavage of Pi09216 and Pi04314 WT during infection.
**Fig. S3** Cleavage of RXLR‐EER effector Pi22926.
**Fig. S4** Secretion of RXLR‐EER effector WT and AAAA‐AAA *in planta*.
**Fig. S5** Detection of RXLR‐EER effector WT and mutants by immunoblotting.
**Fig. S6** Secretion of RXLR‐EER effector AAAA‐EER and RXLR‐AAA *in planta*.
**Fig. S7** LC‐MS/MS analysis of Pi04314AAAA‐EER upper and lower bands.
**Fig. S8** Additional evidence for the cleavage after L in the RXLR motif.
**Fig. S9** Secretion of additional RXLR‐EER effectors *in vitro* and *in planta*.
**Fig. S10** Secretion of Pi04314RXLR‐EER‐RXLR and Pi04314AAAA‐EER‐RXLR *in planta* and *in vitro*.
**Table S1** Primers used in vector construction for *Phytophthora infestans* transformation and *in planta* expression.

**Table S2** LC‐MS/MS identified peptides from the sample immunoprecipitated from the Pi04314 wild‐type culture filtrate (three biological replicates).
Please note: Wiley is not responsible for the content or functionality of any Supporting Information supplied by the authors. Any queries (other than missing material) should be directed to the *New Phytologist* Central Office.

## Data Availability

The data supporting this study’s findings are available within the article and in the Supporting Information (Figs S1–S10; Tables S1, S2).
